# Search for *Mycobacterium avium* Subspecies *paratuberculosis* Antigens for the Diagnosis of Paratuberculosis

**DOI:** 10.1155/2012/860362

**Published:** 2012-06-24

**Authors:** María Laura Mon, Mariana Viale, Guido Baschetti, Fiorella Alvarado Pinedo, Andrea Gioffre, Gabriel Travería, Peter Willemsen, Douwe Bakker, María Isabel Romano

**Affiliations:** ^1^Instituto de Biotecnología, CICVyA, INTA, De Los Reseros y Dr. Nicolás Repetto S/N, Hurlingham, 1686 Buenos Aires, Argentina; ^2^Centro de Diagnóstico e Investigaciones Veterinarias (CEDIVE), Facultad de Ciencias Veterinarias, Universidad Nacional de la Plata (UNLP), B7130AGC Buenos Aires, Argentina; ^3^Department of Bacteriology and Central Veterinary Institute Lelystad, 8221 RA Lolystad, The Netherlands

## Abstract

The aim of this study was to evaluate a wide panel of antigens of *Mycobacterium avium* subsp. *paratuberculosis* (MAP) to select candidates for the diagnosis of paratuberculosis (PTB). A total of 54 recombinant proteins were spotted onto nitrocellulose membranes and exposed to sera from animals with PTB (*n* = 25), healthy animals (*n* = 10), and animals experimentally infected with *M. bovis* (*n* = 8). This initial screening allowed us to select seven antigens: MAP 2513, MAP 1693, MAP 2020, MAP 0038, MAP 1272, MAP 0209c, and MAP 0210c, which reacted with sera from animals with PTB and showed little cross-reactivity with sera from healthy animals and animals experimentally infected with *M. bovis*. The second step was to evaluate the antigen cocktail of these seven antigens by ELISA. For this evaluation, we used sera from animals with PTB (*n* = 25), healthy animals (*n* = 26), and animals experimentally infected with *M. bovis* (*n* = 17). Using ELISA, the cocktail of the seven selected MAP antigens reacted with sera from 18 of the 25 animals with PTB and did not exhibit cross-reactivity with healthy animals and only low reactivity with animals with bovine tuberculosis. The combined application of these antigens could form part of a test which may help in the diagnosis of PTB.

## 1. Introduction

PTB is a prevalent and economically important disease that affects cattle and thus impacts on the cattle industry. It is caused by MAP.

Clinical PTB is characterized by chronic granulomatous enteritis with clinical signs of diarrhea, weight loss, decreased milk production, and mortality. However, most infected cattle show no clinical signs during the prolonged incubation stage of infection [1]. 

On the other hand, a number of theories have proposed that the principal infective agent of Crohn's disease, a chronic enteropathy in humans, is MAP [2–4]. The economic impact and possible link to Crohn's disease highlights the importance of the development of control programs at the herd level. To this end, it is necessary to improve the diagnostic methods of PTB. 

Cattle are most often infected as young calves, before 6 month of age, but some studies have shown that infection may also occur in adult cattle. Fecal shedding of MAP generally starts after 2 years, and clinical symptoms appear after an incubation period of 2–10 years. In addition, the elimination of the agent through the stool is very variable [5]. 

Cell-mediated immune response wanes with progression of the disease and when this occurs, a humoral immune response becomes measurable. It has been shown that cattle are more likely to have a combined antibody and cellular response rather than a switch from cellular to antibody response [6–8]. Among tests to detect serum antibody to MAP, ELISAs are the most widely used. Several commercial ELISA kits for bovine PTB are currently available, and multiple studies have compared their accuracy [9, 10]. Comparative studies of ELISAs with different antigens have shown discrepancies in the ability of these tests to identify all infected animals [11]. Some authors have suggested that this may be due to the lack of representation of the entire range of immunodominant antigens for MAP in a given ELISA test [12]. Then, one of the crucial components of this test is the antigen used for the preparation of the ELISA test. The antigen most widely used for the serological diagnosis of PTB is PPA-3, which is the *M. avium* strain 18 protoplasmic antigen. Currently, antigen-based tests to detect MAP with a mixture of proteins include whole-cell sonicated extract, parcel purified antigen, and protoplasmic antigens. These antigens show variability in potency and cross-reaction. This diagnostic method has drawbacks due to the cross-reaction with animals sensitized with this mycobacterium or other pathogens antigenically related to MAP [11]. Since the sequencing and analysis of the entire MAP genome was obtained [13], several specific proteins have been detected in the genome of MAP and the immunoreactivity of these proteins investigated [14]. Bannantine et al. [15] developed a spot protein array for initial antigen screening. Available diagnostic MAP antigens are reviewed in Mikkelsen et al. [16]. However, individual antigens are able to identify only a subset of PTB-infected animals. Then, a mix of antigens could be a good candidate for serological diagnosis.

In the present work, MAP antigens were obtained after fractionating proteins from the whole cell or membrane or secreted fraction, resolved with two-dimensional gels, printed in line onto nitrocellulose membranes, and analyzed with sera from animals with PTB. 

MAP proteins recognized specifically by sera from PTB-infected animals were used to develop a cocktail of selected antigens to be evaluated by ELISA. 

## 2. Material and Methods

### 2.1. Animals

 Sera from a total of 43 animals from different groups (a) cattle coming from PTB-free herds and with MAP negative fecal cultures (negative control animals) (*n* = 10), (b) cattle naturally infected, with MAP-positive fecal cultures or ELISA-positive tests (*n* = 25), and (c) animals experimentally infected with *M. bovis* with lesions (*n* = 8) were used to evaluate the 54 recombinant proteins of MAP.

The final screening using a cocktail of seven selected antigens was carried out with sera from 68 cattle: 25 cows with MAP positive fecal culture or ELISA-positive tests, 26 cows from negative herds with no suspected cases of PTB and negative tests (fecal cultures, serology and INF-g), and 17 from animals experimentally infected with *M. bovis* and with lesion at the time of necropsy. 

### 2.2. Selection of Antigens

 With the aim of identifing and characterizing immunoreactive proteins for their possible use in diagnosis, we extracted proteins of MAP by treatment of cells with sodium dodecyl sulfate at 50°C [17]. The proteins were resolved by one- or two-dimensional SDS-PAGE, carried out in duplicate. Both duplicates were stained with colloidal Coomassie blue and the other was transferred to a nitrocellulose membrane (Amersham Hybond-ECL, GE Healthcare Life Sciences, Buckinghamshire, UK) to perform a Western blot with a PTB-positive bovine serum sample. Six proteins were immunoreactive with sera from animals with PTB and as nonimmunoreactive with sera from animals infected with *M. avium*. These proteins were cut from the gel and identified by MALDI TOF. This study identified these proteins as encoded by MAP 1962 (Glutamine synthetase), MAP 4143 (Elongation factor tu), MAP 0187c (SodA), MAP 3194, MAP 3205, and MAP 3206, as potential diagnostic antigens. 

The other 48 proteins here evaluated were from CIDC-Lelystad,Central Institute for Animal Disease Control Department Bacteriology, and TSEs, Lelystad, the Netherlands. 

### 2.3. Recombinant Antigens

The 54 proteins were produced as recombinant. Briefly, they were cloned and purified essentially as described previously [18], where all proteins were PCR-cloned using 5′- and 3′-primers amplifying DNA fragments encoding the mature protein except for secreted proteins, which were cloned without the signal peptide. The DNA fragments were cloned into expression vectors. The antigens were cloned into the pET33b (Novagen Inc.,Madison, WI,USA) and pRSET (Invitrogen Corp., Carlsbad, CA, USA) vectors, and expression was induced by the production of T7 RNA polymerase in BL21 (DE3) *E. coli*. These cells also produce T7 lysozyme to reduce the basal expression of the target genes. The secreted antigens described in Willemsem et al. [18] (MAP 2609, MAP 2942c and MAP 0210c) were cloned in pQE80 (Qiagen, Germantown, MD, USA). Antigens were purified using their histidine-tagged N-terminal region and Nickel-affinity columns, (1 mL HisTrap HP columns) (GE Healthcare Life Sciences, UK). Before that, solubilization of the recombinant antigen was established by a buffer containing 6 M Guanidine, 20 mM Tris (pH 8.0), 0.5 M NaCl, or 50 mM Imidazol, 0.25% CHAPS, 1 mM DTT, 0.5 mM PMSF, and 1% iso-propanol to solve proteins from inclusion bodies (Guanidine), reduce nonspecific binding (Imidazol), and decrease the amount of LPS (iso-propanol) or 8 M Urea. After affinity-purification, the antigens were dialyzed (10 kDa cut-off, except for Map 4000c, for which a 1 kDa cut-off was used) against a buffer containing 0 to 6 M Urea, 10 mM Tris (pH 8.0), 25% glycerol, 1 mM DTT, 0.5 mM PMSF, 1% iso-propanol or 0 to 7 M Urea, 10 mM Tris (pH 8.0), 10% glycerol, 1 mM DTT, 0.5 mM PMSF, and 1% iso-propanol. The urea concentration was the minimal concentration needed to keep the proteins solubilized and determined empirically.

The recombinant proteins were analyzed by Coomassie-stained SDS-PAGE to test purity.

### 2.4. Coomassie Blue Staining

**Figure 1:**
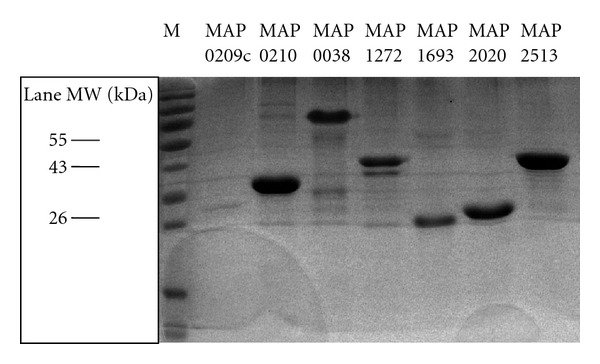
Recombinant proteins analyzed by coomassie-stained SDS-PAGE to test purity.

 A gel containing six of the recombinant purified proteins included in the cocktail is shown in Figure 1. The protein encoded by MAP 0209c (of 56.5 kDa) is shown on the first lane but is not visible because it is very close to the front of the run. The proteins were run in a 15% polyacrylamide gel and then incubated in a Coomassie blue solution 0.05% (Coomassie brilliant blue R250 0.05%, methanol 50%, acid acetic 10%) for 1.5 hours with agitation. The Coomassie blue solution was then removed and bleached with bleaching solution (methanol 50%, acetic acid 10%).

### 2.5. Evaluation of Humoral Response by a Line Print Immunoassay

 The panel of 54 proteins was evaluated as follows: 20 *μ*L of each antigen was applied to a nitrocellulose membrane at a concentration of 100 *μ*g/mL, using a semiautomatic aerosolizer (Camag Scientific Inc., Wilmington, Delaware). The membranes were blocked with 50 mL of blocking solution (5% milk TBS) for 1 h. The membranes were then placed in a “miniblotter” (Isogen BioSolutions, the Netherlands). This allowed parallel analysis of 45 sera. We evaluated serum dilutions of 1 : 100. After 1 h incubation, the serum samples were aspirated and washed three times for 10 min with TBS 1x. The membrane was then incubated for 1 h with protein G conjugated to peroxidase (1 : 1500). The membrane was washed three times for 10 min with TBS 1X and revealed by chemiluminescence (Pierce ECL western blotting, Thermo Scientific, USA). 

Line print immunoassay generated macroarrays of recombinant proteins which were evaluated with sera from animals with PTB. In order to identify potentially cross-reactive proteins, the macroarrays were also used to probe against sera from healthy animals and from animals experimentally infected with *M. bovis*. The macroarrays were subjected to densitometry analysis to provide quantification for reactivity at each spot, reported as spot intensities.

### 2.6. Bioinformatic and Statistical Análisis

 The panel of 54 recombinant proteins included in the present study was characterized *in silico*: molecular weight, location prediction, and homology with other mycobacterial proteins (Table 1). PSORTb analysis was used to predict protein localization based on a number of factors including transmembrane helices, signal peptide, motif search, and similarity to proteins with known subcellular location. (http://www.psort.org/psortb/). According to PSORTb prediction, the set of recombinant proteins contains 28 cytoplasmic proteins, 7 cytoplasmic and membrane proteins, 6 extracellular proteins, and 13 proteins with uncertain localization (Table 1). 

BLAST similarity searches were performed locally on coding sequences by comparison with the GenBank nonredundant protein database (Table 1). 

### 2.7. Measurement of Spot Intensities

 Quantitative spot intensities were obtained by performing a densitometric scan of the membrane. For the analysis of the results, the intensities of the points were measured with the ImageQuant TL Array Version 7.0 Software, (GE Healthcare, Pittsburgh, PA).

This quantification software processes spot intensities on the array and determines the mean intensities of pixels within a spot as well as those of the background pixels around the spot. These local-background intensities are subtracted from the raw signals to obtain the local-background-corrected levels. The measured diameter that was selected for scans of different arrays was consistent for all the arrays in this study. Adjusted intensities were obtained following normalization of each spot. Five proteins (MAP 0038, MAP 1272, MAP 1693, MAP 0210c, and MAP 0209c) showed a stronger mean intensity with sera from MAP-infected animals than with sera from non-MAP-infected animals and were thus selected for a cocktail of antigens (Table 2).

MAP 2020 and MAP 2513 were also selected because although they did not show a strong intensity with sera from MAP-infected animals, they recognized nine and six animals with PTB, respectively (Table 3), and because these proteins had not been previously evaluated or reported in the literature. 

### 2.8. ELISA

 The antigens used for the ELISA test were PPA-3 (Allied Monitor, Inc. USA) and a cocktail with the seven antigens selected (MAP 2513, MAP 1693, MAP 2020, MAP 0038, MAP 1272, MAP 0209c, and MAP 0210c). The cocktail was prepared with 30 *μ*g of each antigen for 1 mL of the mixture. The microtiter plates were coated at 4°C overnight with 100 *μ*L of 40 *μ*g/mL PPA-3 or 20 *μ*g/mL of the cocktail in carbonate buffer (pH: 9.6). Then, the plates were saturated with 100 *μ*L of PBS/0.5% w/v gelatin for 1 h at 37°C, then washed five times with PBS/0.1% Tween20 (PBS/T), and incubated for 1 h at 37°C with 100 *μ*L of 100-fold dilution of sera in PBS/T containing 0.5% (w/v) gelatin. The plates were then washed five times with PBS/T and incubated for 30 min at 37°C with 100 *μ*L of 1500-fold dilution of peroxidase-conjugated protein in PBS/T containing 0.5% (w/v) gelatin. Plates were washed five times with PBS/T, and 50 *μ*L of peroxidase substrate was added. Optical density (OD) was measured at 405 nm.

## 3. Results 

54 proteins were evaluated by Line print immunoassay with sera from healthy animals, animals with PTB, and animals experimentally infected with *M. bovis*. The stronger mean intensity values are listed in Table 2. Serum samples with density values higher than the mean obtained with the control (PBS) were considered positive and the numbers of animals reactive with each protein are shown in Table 3. 

The antigens selected were those that showed stronger intensity with sera from MAP-infected animals than with sera from non-MAP-infected animals. These antigens were MAP 0038, MAP 0210c, MAP 1272, MAP 1693c, and MAP 0209, shown in bold in Tables 2 and 3. In addition, we selected two antigens, MAP 2020 and MAP 2513, because they recognized nine and six animals with PTB (Table 3), respectively, and because they had not been previously evaluated or reported in the literature.

These results contributed to the development of an antigen mixture with seven antigens (MAP 0038, MAP 0210c, MAP 1272, MAP 1693c, MAP 2020, MAP 2513, and MAP 0209c). 

The ORF of MAP 1272 codes for a protein that possesses an NLP/P60 domain of unknown function that is found in several lipoproteins. MAP 0210 codes for the P36/Erp protein of *M. bovis*, which has been studied in our laboratory [19]. PSORTB analysis software predicted that MAP 2513 is localized in the cytoplasm, MAP 0210c in the cytoplasm-membrane, and that MAP 1272 and MAP 0209c are extracellular. The remaining proteins were of unknown localization. All seven of these proteins have not been previously evaluated or reported in the literature, except for MAP 0210c, which has been studied by Willemsen et al. [18].

**Figure 2:**
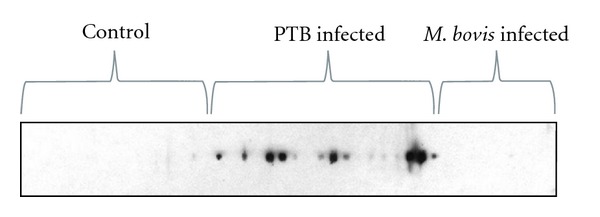
Evaluation of the cocktail by a Line print immunoassay. Result of the cocktail with seven antigens (MAP 0038, MAP 0210c, MAP 1272, MAP 1693, MAP 2020, MAP 0209c and MAP 2513) printed in a membrane of nitrocellulose and evaluated with the sera from healthy animals and animals infected with MAP and *M. bovis*.

The cocktail of seven antigens was printed in the nitrocellulose membrane and evaluated by Line print immunoassay with sera from animals with PTB and healthy controls and animals experimentally infected with *M. bovis* (Figure 2). By line print immunoanalysis, 14 out of 25 of the sera from animals with PTB developed antibody response to the cocktail. This cocktail was not recognized with the sera from animals experimentally infected with *M. bovis* here evaluated and two serum samples from healthy animals gave a very weak signal (Figure 2).

In addition, ELISAs with PPA-3 and with the cocktail with seven antigens were evaluated with sera from animals with PTB (*n* = 25), healthy animals (*n* = 26), and animals experimentally infected with *M. bovis* (*n* = 17). 

**Figure 3:**
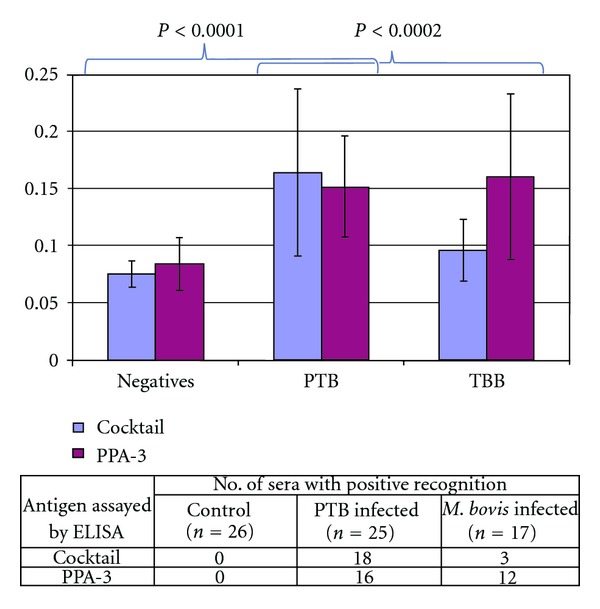
Reactivity of bovine sera to the cocktail by ELISA. Comparison of the diagnostic performance of using PPA-3 (Allied monitor) and the antigen cocktail (MAP 0038, MAP 0210c, MAP 1272, MAP 1693, MAP 2020, MAP 0209c, and MAP 2513).

The ELISA-PPA-3 test recognized 16 out of the 25 animals with PTB (64%) but also 12 of the 17 animals experimentally infected with *M. bovis*, while the ELISA-cocktail detected 18 of 25 animals with PTB (72%) and only 3 of the 17 animals experimentally infected with *M. bovis* (Figure 3). Both ELISAs did not have a reaction with sera from non-infected controls; however, when using sera from *M. bovis*-infected, ELISA-PPA-3 test recognized (12/17) 70,5% of these animals and ELISA-cocktail (3/17) 17,6% of these animals.

This new ELISA for bovine PTB showed 72% of sensitivity and had higher specificity than the ELISA with PPA-3 as antigen, using animals experimentally infected with *M. bovis*.

## 4. Discussion

The early and specific diagnosis of PTB is still a challenge. It has generally been believed that the early immune response to infection with MAP consisted primarily of a cellular immune response characterized by interferon gamma production, and this response would later be replaced by antibody production. However, some studies have shown that antibodies appear much earlier and therefore ELISA could be used as an early diagnostic tool [6–8]. Then it is necessary to characterize MAP antigens to increase the sensitivity and specificity of the ELISA test for PTB diagnosis. The MAP genome sequencing represented a significant advance and will most likely contribute with new tools for diagnosis. The evaluation of a specific panel of antigens such as that studied in the present work is the first step in the selection of candidates to be studied at different herds with PTB of our country. This is an important area, since novel antigens that could improve the diagnosis of MAP-infected cattle are needed.

The proteomic approach has been used to define specific antigens by 2D fraction of MAP proteins by several researchers [20–24]. Another approach to obtain specific antigens is to express recombinant proteins from cloned MAP-coding sequences and use them to construct a protein array [15]. Here we selected MAP proteins by 1D or 2D electrophoresis and developed macroarrays by line print of these proteins. These macroarrays were probed with sera from animals with PTB and healthy controls and animals experimentally infected with *M. bovis*.

After evaluation of 54 proteins of MAP with sera from PTB-infected, experimentally infected with *M. bovis* and healthy animals, we selected seven proteins, which were incorporated in an ELISA to develop an antigen-based diagnostic test (ELISA-cocktail). This new ELISA for bovine PTB showed 72% of sensitivity and had higher specificity than the ELISA with PPA-3 as antigen (Figure 3). This sensitivity was based on 25 animals from MAP fecal culture-positive or ELISA-positive cattle, because naturally infected cattle may represent various stages of MAP infection and serum samples only from culture-positive animals can express differentiated antigen patterns [25]. In addition, the use of only culture-positive animals to estimate the sensitivity of ELISA may increase the values of the test because most culture-positive animals are also ELISA-positive [11].

The results presented here suggest that several specific antigens can improve the detection of MAP infection. In fact, the profiles of antibody response varied considerably and then the antibody response to single antigens was not prominent, while simultaneous usage of several recombinant antigens is able to recognize the ongoing antibody response over time in the course of infection. 

In summary, here we identified novel antigens of MAP by using multiple antigen print immunoassay. Based on this knowledge, we developed an antigen cocktail, which increased the correct diagnosis of MAP-infected animals in comparison with the results of ELISA-PPA-3. The study presents an antigen cocktail, which could be of diagnostic significance for further researches. However, the cocktail needs to be evaluated by larger sample sizes in order to estimate its sensitivity and specificity. In addition, the results here shown with the 54 proteins, indicated that other proteins not included were also good candidates. Then, new cocktails should be incorporated and evaluated to increase the sensitivity and specificity of the ELISA test for diagnosis of PTB. 

## Figures and Tables

**Table 1:** Characterization *in silico* of a panel of 54 recombinant proteins, included in the present study.

Antigen (ORF no.)	Predicted localization	Theoretical MW	Homology with other mycobacteria
MAP 0011	Cytoplasmic	19,196 kDa	Iron-regulated peptidyl-prolyl cis-trans isomerase A in *Mycobacterium tuberculosis* H37Rv
MAP 0034	Cytoplasmic-Membrane	44 kDa	P44 protein in *Mycobacterium avium* subsp. *avium *
MAP 0047c	Extracellular	41,1 kDa	Lpp-LpqN family conserved in Mycobacteriaceae
**MAP 0038**	**Unknown**	**48,7 kDa**	**Hypothetical protein Mb0027 in ** ***Mycobacterium bovis*** ** AF2122/97**
MAP 0187	Extracellular	23 kDa	Superoxide dismutase in *Mycobacterium bovis *
**MAP 0209c**	**Extracellular**	**56,5 kDa**	**Protein potentially involved in peptidoglycan biosynthesis in ** ***Mycobacterium avium*** ** subsp. ** ***avium***
**MAP 0210c**	**Cytoplasmic-Membrane **	**30,7 kDa**	**Secreted antigen P36/P34 precursor in ** ***Mycobacterium bovis*** **, maxima ident 60%**
MAP 0211	Cytoplasmic	46 kDa	UDP-galactopyranose mutase glf in *Mycobacterium tuberculosis *
MAP 0297	Cytoplasmic	55,23 kDa	Hypothetical protein Mb1161 *Mycobacterium bovis* AF2122/97
MAP 0334	Unknown	34,386 kDa	Oxidoreductase in *Mycobacterium bovis* AF2122/97
MAP 0900	Cytoplasmic-Membrane	29,6–34 kDa	Antigen 34 kDa in *Mycobacterium tuberculosis* CDC1551
MAP 0946c	Cytoplasmic-Membrane	33,5 kDa	Sigma factor in *Mycobacterium avium* 104
MAP 1012c	Cytoplasmic	37,374 kDa	Hypothetical protein TMAG_01006 in *Mycobacterium tuberculosis *
MAP 1050c	Cytoplasmic	33 kDa	Peptidyl-prolyl cis-trans isomerase, cyclophilin-type conserved in Mycobacterium
**MAP 1272**	**Extracellular **	**33,4 kDa**	**NLP/P60 family protein in ** ***Mycobacterium tuberculosis*** ** CDC1551**
MAP 1293	Unknown	49,24 kDa	Histidinol dehydrogenase his D in *Mycobacterium tuberculosis *
MAP 1308	Unknown (This protein may have multiple localization sites.)	46 kDa	Prolipoprotein diacylglyceryl transferase lgt in *Mycobacterium tuberculosis *
MAP 1564c	Unknown	23,01 kDa	Short chain dehydrogenase in *Mycobacterium bovis *
MAP 1589c	Cytoplasmic	21,60 KDa	Alkyl hydroperoxide reductase subunit C in *Mycobacterium tuberculosis *
MAP 1653	Unknown	16,7–20 kDa	Thiol peroxidase tpx in *Mycobacterium tuberculosis* T17
**MAP 1693c**	**Unknown **	**18,30 kDa**	**Peptidyl-prolyl cis-trans isomerase domain-containing protein conserved in Mycobacterium**
MAP 1754c	Cytoplasmic-Membrane	30,84 kDa	Hypothetical protein Rv2005c in *Mycobacterium tuberculosis* H37Rv
MAP 1889c	Cytoplasmic	28 kDa	Wag31 protein in *Mycobacterium avium* 104
MAP 1962	Cytoplasmic	53,68 kDa	Glutamine synthetase glnA1 in *Mycobacterium tuberculosis *
**MAP 2020**	**Unknown**	**26,90 kDa**	**Cutinase in ** ***Mycobacterium bovis*** ** AF2122/97**
MAP 2167	Extracellular	17 kDa	Low molecular weight protein antigen cfp2 in *Mycobacterium tuberculosis *
MAP 2182	Cytoplasmic-Membrane	16 kDa	Deazaflavin-dependent nitroreductase family protein in Mycobacterium
**MAP 2513**	**Cytoplasmic **	**36,50 kDa**	**Alkanal monooxygenase alpha chain in *Mycobacterium avium* 104**
MAP 2609	Cytoplasmic-Membrane	11,40 kDa	Low molecular weight T-cell antigen TB8.4 in *Mycobacterium tuberculosis *
MAP 2676c	Cytoplasmic	13,89 kDa	Hypothetical protein MAV_1246 in *Mycobacterium avium* 104
MAP 2685	Unknown	21,20 kDa	Hypothetical protein BCG_1169c in *Mycobacterium bovis* BCG str. Pasteur 1173P2
MAP 2878c	Cytoplasmic	25,43 kDa	Dihydrodipicolinate reductase in *Mycobacterium bovis* BCG str. Pasteur 1173P2
MAP 2942c	Extracellular	18,30 kDa	Soluble secreted antigen MPT53 in *Mycobacterium tuberculosis *
MAP 2956	Cytoplasmic	30,02 kDa	30S ribosomal protein S2 in *Mycobacterium tuberculosis* H37Rv
MAP 3175c	Cytoplasmic	41,38 kDa	Peptide chain release factor 2 in *Mycobacterium tuberculosis* H37Rv
MAP 3194	Cytoplasmic	30,46 kDa	Pyruvate carboxyl transferase in *Mycobacterium avium *
MAP 3205	Cytoplasmic	27 kDa	nuoE NADH dehydrogenase subunit E in *Mycobacterium avium* 104
MAP 3206	Cytoplasmic	48-49 kDa	nuoF NADH-Quinone oxidoreductase subunit F in *Mycobacterium tuberculosis *
Mb 3341c	Unknown	10,63 kDa	Equivalent to Rv3312A, len: 103 aa, from *Mycobacterium tuberculosis* strain H37Rv
MAP 3402	Cytoplasmic	33,28 kDa	Thiosulfate sulfurtransferase in *Mycobacterium * *bovis* AF2122/97
MAP 3457	Cytoplasmic	47,61 kDa	O-acetylhomoserine sulfhydrylase metC in *Mycobacterium tuberculosis* T92
MAP 3491	Cytoplasmic	28,16 kDa	Hydrolase in *Mycobacterium tuberculosis* SUMu003
MAP 3527	Unknown	35,70 kDa	Serine protease PepA in Mycobacterium tuberculosis H37Rv
MAP 3627	Cytoplasmic	37,37 kDa	O-methyltransferase in *Mycobacterium tuberculosis* H37Rv
MAP 3651	Cytoplasmic	44 kDa	Acyl-CoA dehydrogenase fadE3 in *Mycobacterium bovis* BCG
MAP 3692	Cytoplasmic	47 kDa	fabG 3-ketoacyl-ACP reductase in *Mycobacterium tuberculosis* H37Rv
MAP 3840	Cytoplasmic	67 kDa	Heat shock protein 70, molecular chaperone DnaK in Mycobacterium
MAP 3841	Unknown	23,57 kDa	Heat shock protein GrpE in *Mycobacterium bovis* AF2122/97
MAP 3857	Cytoplasmic	18,73 kDa	Orotate phosphoribosyltransferase phosphoribosyltransferase in *Mycobacterium tuberculosis* CDC1551
MAP 3936	Cytoplasmic	57 kDa	Heat shock protein 65, GroEL in Mycobacterium sp
MAP 4000c	Unknown	12 kDa	Esat-6 like protein esxF in Mycobacterium and hypothetical protein Mb3935c in *Mycobacterium bovis *
MAP 4143	Cytoplasmic	43,77 kDa	Iron-regulated elongation factor tu in *Mycobacterium tuberculosis *
MAP 4147	Cytoplasmic	42,09 kDa	Ferredoxin reductase in *Mycobacterium tuberculosis* H37Rv
MAP 4227c	Cytoplasmic	30,14 kDa	Hypothetical protein Rv3463 in *Mycobacterium tuberculosis* H37Rv

**Table 2:** Spot intensities for protein macroarrays.

Antigen assayed	Mean spot intensity of proteins exposed to sera		
	control (*n *= 10)	PTB infected (*n *= 25)	TB infected (*n *= 8)
MAP 0011	112	135	0
MAP 0034	240	247	0
**MAP 0038**	**141**	**860**	**0**
MAP 0047	344	309	0
MAP 0187c	184	0	0
**MAP 0209c**	**367**	**499**	**0**
**MAP 0210c**	**0**	**398**	**0**
MAP 0211	*0*	*572*	*0*
MAP 0297	117	225	130
MAP 0334	108	167	1
MAP 0900	*0*	*749*	*0*
MAP 0946	*218*	*900*	*295*
MAP 1012	280	0	0
MAP 1050	268	275	0
**MAP 1272**	**223**	**733**	**0**
MAP 1293	585	481	396
MAP 1308	163	894	0
MAP 1564	259	344	0
MAP 1589c	323	682	466
MAP 1653	398	844	461
**MAP 1693**	**226**	**967**	**209**
MAP 1754	0	142	27
MAP 1889c	280	180	494
MAP1962	341	270	354
**MAP 2020**	**0**	**150**	**228**
MAP 2167	315	0	0
MAP 2182c	223	117	192
**MAP 2513**	**41**	**164**	**291**
MAP 2609	137	308	0
MAP 2676	201	138	0
MAP 2685	317	323	0
MAP 2878	307	323	0
MAP 2942	136	251	0
MAP 2956	345	119	424
MAP 3175	343	130	0
MAP 3194	0	100	0
MAP 3205	0	0	0
MAP 3206	100	200	0
Mb 3341	159	171	178
MAP 3402	205	178	250
MAP 3457	447	365	325
MAP3491	286	454	177
MAP 3527	528	414	635
MAP 3627	207	265	0
MAP 3651	1000	906	958
MAP 3692c	305	212	0
MAP 3840	0	198	712
MAP 3841	707	678	457
MAP 3857	373	267	265
MAP 3936	381	544	691
MAP 4000c	134	242	0
MAP4143	413	397	439
MAP 4147	1000	556	0
MAP 4227	138	101	0
PPDA	208	105	256
PPDB	346	531	1468
PPA-3	131	609	108
**Cocktail**	**140**	**1460**	**0**

Intensities were obtained using ImageQuant TL Array Version 7.0 Software, (GE Healthcare, Pittsburgh, PA). Antigens selected to evaluate as cocktail are shown in bold. PPA-3: Paratuberculosis protoplasmatic antigen (Allied Monitor, Inc. USA); PPDA and PPDB: avian- and bovine-derivative protein purified, respectively (Prionic Switzerland). Cocktail: mix of the 7 antigens shown in bold.

**Table 3:** Reactivity of bovine sera to the panel of selected antigens from MAP.

Antigen assayed	No. of sera with positive recognition		
	control (*n* = 10)	PTB infected (*n* = 25)	TB infected (*n* = 8)
MAP 0011	0	5	4
MAP 0034	5	5	0
**MAP 0038**	**3**	**8**	**0**
MAP 0047	3	4	0
MAP 0187c	1	1	0
**MAP 0209c**	**4**	**9**	**0**
**MAP 0210c**	**0**	**8**	**0**
MAP 0211	0	6	0
MAP 0297	2	4	1
MAP 0334	2	15	1
MAP 0900	0	3	0
MAP 0946	1	8	0
MAP 1012	2	0	0
MAP 1050	4	9	0
**MAP 1272**	**2**	**8**	**0**
MAP 1293	10	13	1
MAP 1308	2	2	0
MAP 1564	5	1	0
MAP 1589c	2	9	2
MAP 1653	10	20	8
**MAP 1693**	**3**	**12**	**2**
MAP 1754	0	5	4
MAP 1889c	7	7	3
MAP 1962	3	16	5
**MAP 2020**	**0**	**9**	**2**
MAP 2167	3	0	0
MAP 2182c	5	1	2
**MAP 2513**	**1**	**6**	**2**
MAP 2609	2	4	0
MAP 2676	1	4	0
MAP 2685	10	2	0
MAP 2878	10	2	0
MAP 2942	2	7	0
MAP 2956	1	1	1
MAP 3175	4	4	4
MAP 3194	0	1	0
MAP 3205	0	0	0
MAP 3206	1	5	0
Mb 3341	4	6	3
MAP 3402	2	20	1
MAP 3457	10	8	4
MAP 3491	7	7	1
MAP 3527	6	25	8
MAP 3627	3	4	0
MAP 3651	10	14	8
MAP 3692c	5	8	0
MAP 3840	0	3	3
MAP 3841	10	14	1
MAP 3857	10	4	4
MAP 3936	5	8	4
MAP 4000c	3	5	0
MAP 4143	8	16	7
MAP 4147	1	3	0
MAP 4227	1	1	1
PPDA	6	1	4
PPDB	4	16	3
PPA-3	1	18	2
**Cocktail**	**2**	**14**	**0**

20 uL of antigens were applied to a nitrocellulose membrane (Amersham Hybond-ECL, GE Healthcare Life Sciences, UK) at a concentration of 100 ug/mL, using a semiautomatic aerolizer (Camag Scientific Inc., Wilmington, Delaware). Membranes were evaluated by immunoblotting using sera from healthy and infected animals. Numbers of sera with antibody response are indicated. Samples with intensities values higher than the media obtained with the control (PBS) were considered positive. Antigens selected to evaluate as cocktail are shown in bold. PPA-3: Paratuberculosis protoplasmatic antigen (Allied Monitor, Inc. USA); PPDA and PPDB: avian- and bovine-derivative protein purified (Prionic Switzerland ); Cocktail: Mix of the 7 antigens shown in bold.

## References

[1] Tiwari A, VanLeeuwen JA, McKenna SLB, Keefe GP, Barkema HW (2006). Johne’s disease in Canada part I: clinical symptoms, pathophysiology, diagnosis, and prevalence in dairy herds. *The Canadian Veterinary Journal*.

[2] Chiodini RJ, Rossiter CA (1996). Paratuberculosis: a potential zoonosis?. *The Veterinary Clinics of North America. Food Animal Practice*.

[3] Hermon-Taylor J, Bull TJ, Sheridan JM (2000). *Mycobacterium avium* subspecies *paratuberculosis* in the causation of Crohn’s disease. *World Journal of Gastroenterology*.

[4] Hermon-Taylor J, Bull TJ, Sheridan JM, Cheng J, Stellakis ML, Sumar N (2000). Causation of Crohn’s disease by *Mycobacterium avium* subspecies *paratuberculosis*. *Canadian Journal of Gastroenterology*.

[5] Sockett DC (1996). Johne’s disease eradication and control: regulatory implications. *The Veterinary Clinics of North America. Food Animal Practice*.

[6] Koets AP, Rutten VPMG, de Boer M, Bakker D, Valentin-Weigand P, van Eden W (2001). Differential changes in heat shock protein-, lipoarabinomannan-, and purified protein derivative-specific immunoglobulin G1 and G2 isotype responses during bovine *Mycobacterium avium* subsp. *paratuberculosis* infection. *Infection and Immunity*.

[7] Waters WR, Miller JM, Palmer MV (2003). Early induction of humoral and cellular immune responses during experimental *Mycobacterium avium* subsp. *paratuberculosis* infection of calves. *Infection and Immunity*.

[8] Begg DJ, de Silva K, Carter N, Plain KM, Purdie A, Whittington RJ (2011). Does a th1 over th2 dominancy really exist in the early stages of *Mycobacterium avium* subspecies *paratuberculosis* infections?. *Immunobiology*.

[9] Fry MP, Kruze J, Collins MT (2008). Evaluation of four commercial enzyme-linked immunosorbent assays for the diagnosis of bovine paratuberculosis in Chilean dairy herds. *Journal of Veterinary Diagnostic Investigation*.

[10] van Schaik G, Haro F, Mella A, Kruze J (2007). Bayesian analysis to validate a commercial ELISA to detect paratuberculosis in dairy herds of Southern Chile. *Preventive Veterinary Medicine*.

[11] Costanzo G, Pinedo FA, Mon ML (2012). Accuracy assessment and screening of a dairy herd with paratuberculosis by three different ELISAs. *Veterinary Microbiology*.

[12] Sugden EA, Stilwell K, Michaelides A (1997). A comparison of lipoarabinomannan with other antigens used in absorbed enzyme immunoassays for the serological detection of cattle infected with *Mycobacterium paratuberculosis*. *Journal of Veterinary Diagnostic Investigation*.

[13] Li L, Bannantine JP, Zhang Q (2005). The complete genome sequence of *Mycobacterium avium* subspecies *paratuberculosis*. *Proceedings of the National Academy of Sciences of the United States of America*.

[14] Hughes V, Bannantine JP, Denham S (2008). Immunogenicity of proteome-determined *Mycobacterium avium* subsp. *paratuberculosis*-specific proteins in sheep with paratuberculosis. *Clinical and Vaccine Immunology*.

[15] Bannantine JP, Waters WR, Stabel JR (2008). Development and use of a partial *Mycobacterium avium* subspecies *paratuberculosis* protein array. *Proteomics*.

[16] Mikkelsen H, Aagaard C, Nielsen SS, Jungersen G (2011). Review of *Mycobacterium avium* subsp. *paratuberculosis* antigen candidates with diagnostic potential. *Veterinary Microbiology*.

[17] Hirschfield GR, McNeil M, Brennan PJ (1990). Peptidoglycan-associated polypeptides of *Mycobacterium tuberculosis*. *Journal of Bacteriology*.

[18] Willemsen PTJ, Westerveen J, Dinkla A, Bakker D, van Zijderveld FG, Thole JER (2006). Secreted antigens of *Mycobacterium avium* subspecies *paratuberculosis* as prominent immune targets. *Veterinary Microbiology*.

[19] Bigi F, Gioffré A, Klepp L (2005). Mutation in the P36 gene of *Mycobacterium bovis* provokes attenuation of the bacillus in a mouse model. *Tuberculosis*.

[20] Cho D, Collins MT (2006). Comparison of the proteosomes and antigenicities of secreted and cellular proteins produced by *Mycobacterium paratuberculosis*. *Clinical and Vaccine Immunology*.

[21] Cho D, Sung N, Collins MT (2006). Identification of proteins of potential diagnostic value for bovine paratuberculosis. *Proteomics*.

[22] Shin SJ, Cho D, Collins MT (2008). Diagnosis of bovine paratuberculosis by a novel enzyme-linked immunosorbent assay based on early secreted antigens of *Mycobacterium avium* subsp. *paratuberculosis*. *Clinical and Vaccine Immunology*.

[23] Hughes V, Smith S, Garcia-Sanchez A, Sales J, Stevenson K (2007). Proteomic comparison of *Mycobacterium avium* subspecies *paratuberculosis* grown in vitro and isolated from clinical cases of ovine paratuberculosis. *Microbiology*.

[24] Leroy B, Roupie V, Noël-Georis I (2007). Antigen discovery: a postgenomic approach to paratuberculosis diagnosis. *Proteomics*.

[25] Radosevich TJ, Reinhardt TA, Lippolis JD, Bannantine JP, Stabel JR (2007). Proteome and differential expression analysis of membrane and cytosolic proteins from *Mycobacterium avium* subsp. *paratuberculosis* strains K-10 and 187. *Journal of Bacteriology*.

